# Exposure assessment of extended-spectrum beta-lactamases/AmpC beta-lactamases-producing *Escherichia coli* in meat in Denmark

**DOI:** 10.3402/iee.v4.22924

**Published:** 2014-02-05

**Authors:** Luís P. Carmo, Liza R. Nielsen, Paulo M. da Costa, Lis Alban

**Affiliations:** 1 Department of Aquatic Production, Abel Salazar Institute for the Biomedical Science (ICBAS), University of Porto, Porto, Portugal; 2 Population Biology Group, Department of Large Animal Sciences, University of Copenhagen, Copenhagen, Denmark; 3 Laboratory of Nutrition, Growth and Quality of Fish, Interdisciplinary Center for Marine and Environmental Research (CIIMAR), University of Porto, Porto, Portugal; 4 Department for Food Safety and Veterinary Issues, Danish Agriculture & Food Council, Copenhagen, Denmark

**Keywords:** E. coli, ESBL, AmpC, exposure assessment, meat

## Abstract

**Introduction:**

Extended-spectrum beta-lactamases (ESBL) and AmpC beta-lactamases (AmpC) are of concern for veterinary and public health because of their ability to cause treatment failure due to antimicrobial resistance in *Enterobacteriaceae*. The main objective was to assess the relative contribution (RC) of different types of meat to the exposure of consumers to ESBL/AmpC and their potential importance for human infections in Denmark.

**Material and methods:**

The prevalence of each genotype of ESBL/AmpC-producing *E. coli* in imported and nationally produced broiler meat, pork and beef was weighted by the meat consumption patterns. Data originated from the Danish surveillance program for antibiotic use and antibiotic resistance (DANMAP) from 2009 to 2011. DANMAP also provided data about human ESBL/AmpC cases in 2011, which were used to assess a possible genotype overlap. Uncertainty about the occurrence of ESBL/AmpC-producing *E. coli* in meat was assessed by inspecting beta distributions given the available data of the genotypes in each type of meat.

**Results and discussion:**

Broiler meat represented the largest part (83.8%) of the estimated ESBL/AmpC-contaminated pool of meat compared to pork (12.5%) and beef (3.7%). CMY-2 was the genotype with the highest RC to human exposure (58.3%). However, this genotype is rarely found in human infections in Denmark.

**Conclusion:**

The overlap between ESBL/AmpC genotypes in meat and human *E. coli* infections was limited. This suggests that meat might constitute a less important source of ESBL/AmpC exposure to humans in Denmark than previously thought – maybe because the use of cephalosporins is restricted in cattle and banned in poultry and pigs. Nonetheless, more detailed surveillance data are required to determine the contribution of meat compared to other sources, such as travelling, pets, water resources, community and hospitals in the pursuit of a full source attribution model.

Extended-spectrum beta-lactamases (ESBL) were defined by the EFSA Panel on Biological Hazards (BIOHAZ) as plasmid-encoded enzymes found in the bacterial family *Enterobacteriaceae*, commonly in *Escherichia coli* and *Klebsiella pneumonia*. ESBL confer resistance to a variety of beta-lactam antibiotics, including penicillins; second-, third-, and fourth-generation cephalosporins; and monobactams (1). The BIOHAZ panel also stated that AmpC beta-lactamases (AmpC) are intrinsic cephalosporinases found on the chromosomal DNA of many gram-negative bacteria. However, the number of AmpC enzymes that are plasmid borne is increasing (1). An example of such plasmidic AmpC is CMY-2.

## Burden of disease

The World Health Organization (WHO) estimated 25,000 deaths each year in the European Union (EU) due to infections with antibiotic-resistant bacteria (2). The increase of antimicrobial resistance caused by ESBL/AmpC is particularly worrying due to their resistance to third- and fourth-generation cephalosporins, which have been considered critically important to human medicine (3).

Infections caused by ESBL/AmpC-producing bacteria are increasing worldwide (4). The European Antimicrobial Resistance Surveillance Network (EARS-Net) stated that no country among the 28 countries reporting to this program has demonstrated decreasing trends of third-generation cephalosporin-resistant *E. coli* over the past few years (5). Moreover, in 2011, more than half of the countries reported proportions between 85 and 100% of third-generation cephalosporin-resistant *E. coli* to be ESBL-producing (5). In Denmark, a steady increase of third-generation cephalosporin-resistant *E. coli* from human bloodstream infections has been observed from 2005 (1.1%) through 2011 (8.5%) (6).

The importance of the increase of ESBL/AmpC among *E. coli* septicemias can also be reflected in the augmented case fatality risk (7), although it should be noted that these bacteraemic cases are often associated with a critical underlying disease, delay in appropriate treatment and older age (8).

In general, the economic burden of antimicrobial resistance is significant (9). Moreover, ESBL/AmpC resistance leads to an increase in the use of carbapenems (4). The use of this class of drugs is related to the emergence of carbapenemases (10), which have the ability to hydrolyze all the beta-lactams and are therefore of concern (1).

## Possible ESBL/AmpC-producing bacteria sources

There are several potential ESBL/AmpC-producing bacteria sources that can be summarized in five major groups: foodborne, direct animal-to-human transmission, human-to-human transmission, environment and infections obtained abroad during travelling. Travelling is considered a source by itself since surveillance programs are normally set at a national level. Moreover, it is more difficult to unveil the specific source when infection is acquired abroad. To our knowledge, the true importance of each of these possible sources for ESBL/AmpC dissemination and their relative relevance for human cases is unknown. Recently, a possible link to food and food-producing animals was made (1), stressing the importance of scrutinizing the role of food animal products to human ESBL/AmpC-producing bacterial infections.

## The Danish situation

Denmark, like other Nordic countries, has always been considered to have a low prevalence of antimicrobial resistance in the livestock population, due to the conscious use and restrictive measures related to antibiotics. The ‘Yellow card’ initiative (11) and the voluntary ban on the use of cephalosporin in pigs (12) are examples of the precautionary Danish policy regarding the antimicrobial use in animal production.

From 2000 to 2008, DANMAP reports consistently showed a very low prevalence of third-generation cephalosporin-resistant bacteria both in live animals and in Danish meat (below 2% of positive samples) (13–21). Meat samples with 5 g of weight were collected at wholesale and retail outlets and a non-selective culture method was used.

To detect ESBL/AmpC-producing bacteria, an antibiotic enriched culture method is preferred (1). In 2009, the first surveillance study using an enriched culture method was performed, followed by a genetic background investigation (22). In relation to the sample weight and place of sample collection, the original procedure was kept. Since then, this protocol has been repeated yearly, revealing an increasing prevalence of ESBL/AmpC-producing *E. coli* in Danish broiler meat from 3% in 2009 (data not published) to 44% in 2011 (23). In the same period, the prevalence in imported broiler meat ranged from 36 to 50% (22–24). This has raised concern that meat may be an increasingly important source of human cases in Denmark.

Hence, the aim of this study was to determine the relative contribution (RC) of various meat types to the exposure of Danish meat consumers to ESBL/AmpC-producing *E. coli* and their potential importance for human infections in Denmark.

## Material and methods

A relative exposure assessment was performed. The approach was to compare the distributions of genotypes in humans and the most common meat sources in Denmark.

### Meat consumption patterns

**Fig. 1:**
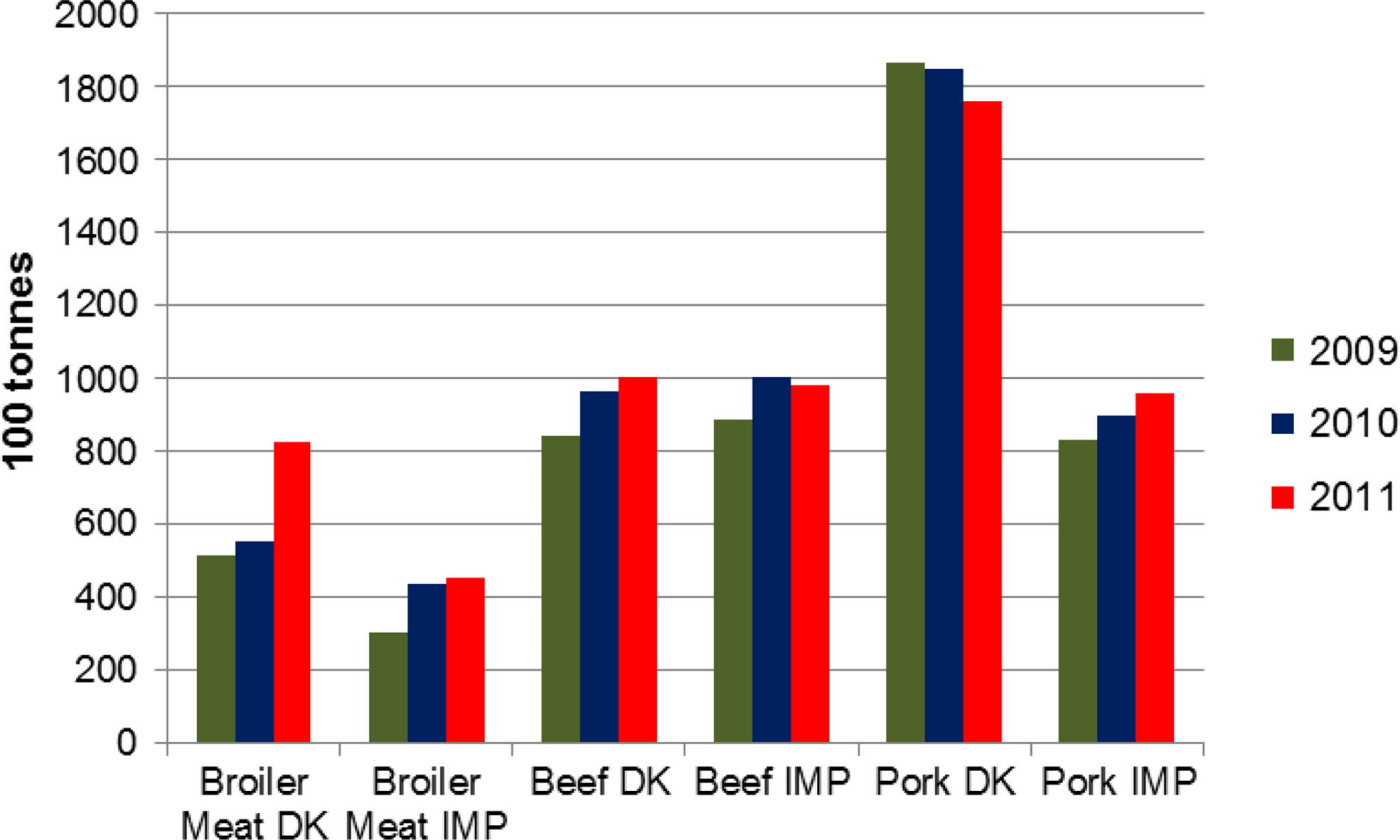
Fresh and frozen meat available for consumption in Denmark of Danish (DK) and imported (IMP) origin between 2009 and 2011. Source: DTU 2011.

Data about the fresh and frozen meat available for consumption in 2009, 2010 and 2011 from the ‘Annual Report on Zoonoses in Denmark 2011’ were consulted and divided into six categories: imported and domestically produced broiler meat, pork and beef (25) (Fig. 1). Consumption was calculated as:

Domestically produced – exported +imported (25).

### ESBL/AmpC-producing *E. coli* occurrence in meat

The data describing the ESBL/AmpC prevalence were collected from the Danish surveillance program for antibiotic use and antibiotic resistance (22–24). In the DANMAP program, meat samples (5 g per sample for all three species) are collected at wholesale/retail level and detection of ESBL/AmpC-producing *E. coli* is conducted using a selective culture method using antimicrobials. This is a qualitative method with a very high sensitivity.

### RC of each type of meat to human exposure

To calculate the contribution of each type of meat and each genotype to human exposure, the prevalence of ESBL/AmpC genotypes was weighted by the amount of meat available for consumption: Weighted Prevalence (WP)=Prevalence * Meat available for consumption. All the WPs were added up to obtain the total pool of ESBL/AmpC in meat (TP). Finally, the WP of each genotype was divided by the ESBL/AmpC Total Pool: RC=WP/TP.

Due to the low number of positive samples for pork and beef and the low number of samples tested in general, the 3 years of sampling were analyzed collectively. However, the described procedure was also applied to each year separately.

### Uncertainty distributions

Using the software @Risk 4.5 Palisade Corporation^®^, beta distributions were created to assess the 95% credibility interval (CI) for each genotype. The main objective of this procedure was to conduct an uncertainty analysis and hereby estimate how high the true prevalence could be given the available data. Beta distributions were used in previous risk assessments to take into account the uncertainty and variability of estimates (26).

The beta distributions were made through a simulation using 1,000,000 iterations. The distributions were defined by (*s*+1, *n−s*+1), where *s* represents the number of positive samples for each genotype in each type of meat from 2009 to 2011, and *n* represents the total number of samples tested for ESBL/AmpC-producing *E. coli* in each type of meat within the same 3-year period.

This procedure was of particular relevance because some genotypes have not been found in the Danish sampling surveillance, but still may have been present in meat and could have played a role for ESBL/AmpC-producing *E. coli* human cases. Their distribution was set as (0+1, *n−*0+1). Some genotypes were not found in meat during the surveillance program, but were found in live animals. The higher the number of samples tested and found negative, the lower the maximum prevalence that could be in the meat.

## Results

### RC of each type of meat to human exposure

**Fig. 2:**
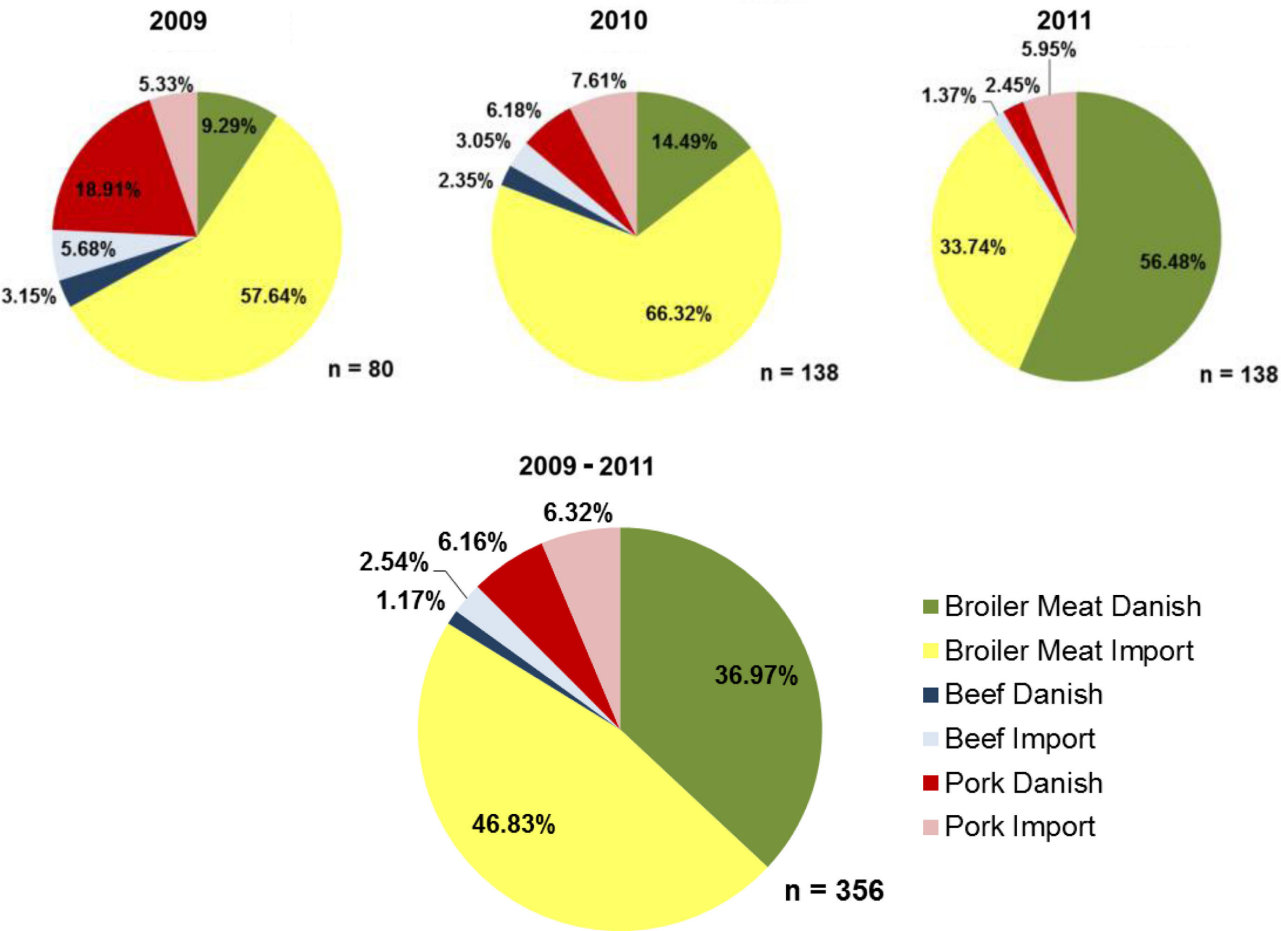
Relative contribution of each type of meat and their respective origin to human ESBL/AmpC- producing *E. coli* exposure, based on Danish data from 2009 to 2011 (Danmap, 2009, 2010, 2011).

During the period 2009–2011, broiler meat contributed the most to the total human exposure to ESBL/AmpC representing 83.8% of the total exposure. Danish broiler meat contributed with 37.0% and imported broiler meat with 46.8%. Danish and imported pork constituted 6.2 and 6.3% of the ESBL/AmpC positive meat available for consumption, respectively. Beef was less important, representing 1.2% in beef of Danish origin and 2.5% of imported origin. The RC of Danish broiler meat increased markedly from 2009 to 2011, while the RC of pork and imported broiler meat decreased in the same period (Fig. 2).

The RC of each genotype varied among the 3 years (Table 1). Most important was the increase in the role of CMY-2 in Danish broiler meat from 4.7% in 2009 to 11.8% in 2010, increasing to 53.5% of the total exposure in 2011. Moreover, CMY-2 constituted the genotype that meat consumers were most exposed to (58.3% across the 3-year period). Broiler meat was the source that contributed the most to this specific exposure (56.8% from broiler meat, 0.8% from pork, and 0.7% from beef).

**Table 1:** Relative contribution (RC) of each type of meat for human exposure considering the origin and the genotypes found in DANMAP surveillance from 2009 to 2011

	RC for human exposure (%)					RC for human exposure (%)					RC for human exposure (%)			
					
Meat type	2009	2010	2011	2009–2011	Origin	2009	2010	2011	2009–2011	Genotype	2009	2010	2011	2009–2011
Pork	24.2	13.8	8.4	12.5						CTX-M-1	0.0	6.2	2.5	3.1
					Danish	18.9	6.2	2.5	6.2	CTX-M-2	12.9	0.0	0.0	2.1
										Others^1^	6.0	0.0	0.0	1.0
										CTX-M-1	2.7	7.6	4.5	5.1
					Imported	5.3	7.6	6.0	6.3	CMY-2	0.0	0.0	1.5	0.8
										CTX-M-14	2.7	0.0	0.0	0.4
Beef	8.8	5.4	1.4	3.7	Danish	3.2	2.4	0.00	1.8	CMY-2	0.0	2.4	0.0	0.7
										Others^2^	3.2	0.0	0.0	0.5
					Imported	5.7	3.1	1.4	2.5	CTX-M-1	5.7	3.1	1.4	2.5
Broiler meat	67.0	80.8	90.2	83.8	Danish	9.3	14.5	56.5	37.0	CMY-2	4.7	11.8	53.5	33.8
										CTX-M-1	4.7	2.7	3.0	3.2
					Imported	57.6	66.3	33.7	46.8	CMY-2	27.6	24.7	20.8	23.0
										CTX-M-1	14.2	29.3	7.9	15.0
										SHV-12	9.2	6.5	4.1	5.6
										Others^3^	5.0	4.1	1.0	2.5
										CTX-M-2	1.6	1.7	0.0	0.8

1 Unknown genotypes (i.e. when it was impossible to determine a specific genotype).

2 TEM-52 and unknown genotypes.

3 TEM-20, TEM-52, up-regulated AmpC and unknown genotypes.

CTX-M-1 was the most frequently isolated genotype in pork and beef at 8.2 and 2.5% of the total exposure, respectively. Including broiler meat, CTX-M-1 represented 28.8% of the total ESBL/AmpC-positive meat on the Danish market from 2009 to 2011.

Considering the origin of positive meat for ESBL/AmpC, a major shift occurred from 2010 to 2011. Both in 2009 and 2010, Danish meat represented less than one third of the ESBL/AmpC meat contributing to human exposure (31.4 and 23.0%, respectively). In 2011, the RC for human exposure of the Danish meat rose to 58.9% and the imported meat declined to 41.1%. However, across the 3-year period, imported meat still contributed the most to human exposure (55.7%).

### Genotype overlapping between meat and human infections

**Fig. 3:**
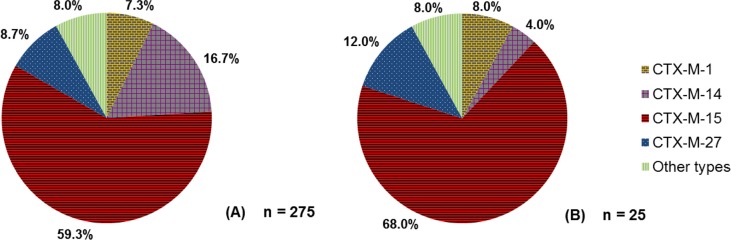
(A) Proportion of various genotypes detected in ESBL-producing *E. coli* urinary tract infections in humans in Denmark in 2011. (B) Proportion of various genotypes detected in ESBL-producing *E. coli* bloodstream infections in humans in Denmark in 2011. Source: DANMAP 2011.

In humans, the so-called ‘pandemic’ CTX-M-15 genotype dominated in 2011 in Denmark, both in blood and urine ESBL-producing *E. coli* isolates, with 68.0 and 59.3%, respectively (23) (Fig. 3). CTX-M-1 was detected in 7.3% of urine and 8.0% of blood isolates (23).

CMY-2 was detected in human infections, but it was not possible to calculate its relative prevalence in urinary tract infections and septicemias. Nevertheless, the number of positive samples was low (data not published).

Considering both the human data and the results from the RC of each genotype to the human exposure through meat, the genotype overlapping between the two reservoirs is low.

### Prevalence estimations for each genotype

CTX-M-15 was the genotype most commonly found in human cases. It was found on rare occasions in Danish pigs and cattle at slaughter: two positive samples in 2009 (*n*=786) and one in 2011 (*n*=777) in pigs, and one positive sample in 2010 (*n*=192) and another in 2011 (*n*=186) in cattle. However, this genotype had not been detected in meat samples previously. Through our calculations, this ESBL gene was estimated to be present in Danish pork and beef with prevalences below 0.7 and 1.0%, respectively, given the available data (Table 2).

**Table 2:** Lower limit, mode and upper limit of prevalence calculated for each genotype in each type of meat through beta distribution with a 95% CI

							Beta distribution of genotype prevalence			
								
Meat type	Origin	Total number of samples for each type of meat *n*	Number of positive samples for each type of meat	Prevalence 2009–2011 (%)	Genotype	Number of positive samples for each genotype *s* (prevalence,%)	Lower limit of prevalence (%)	Mode of prevalence (%)	Upper limit of prevalence (%)	Detected in humans (DANMAP 2011)
Pork	Danish	562	7	1.2	CTX-M-1	4 (0.7)	0.4	0.7	1.8	Yes
					CTX-M-2	2 (0.4)	0.2	0.4	1.2	No
					Unknown	1 (0.2)	0.0	0.2	0.9	N/A^1^
					Others	0 (0.0)	0.0	0.0	0.7	N/A^2^
	Imported	448	11	2.4	CTX-M-1	9 (2.0)	1.1	2.0	3.8	Yes
					CTX-M-14	1 (0.2)	0.0	0.2	1.3	Yes
					CMY-2	1 (0.2)	0.0	0.2	1.3	Yes^3^
					Others	0 (0.0)	0.0	0.0	0.9	N/A
Beef	Danish	382	2	0.5	TEM-52	1 (0.3)	0.0	0.3	1.3	No
					CMY-2	1 (0.2)	0.0	0.3	1.3	N/A
					Others	0 (0.0)	0.0	0.0	1.0	N/A
	Imported	298	3	1.1	CTX-M-1	3 (1.1)	0.3	1.0	3.0	Yes
					Others	0 (0.0)	0.0	0.0	1.4	N/A
Broiler meat					CMY-2	70 (16.0)	12.7	16.1	19.8	Yes
	Danish	440	78	17.8	CTX-M-1	8 (1.8)	0.9	1.8	3.6	Yes
					Others	0 (0.0)	0.0	0.0	0.9	N/A
					CMY-2	120 (21.0)	17.9	21.0	24.5	Yes
					CTX-M-1	84 (14.7)	12.1	14.5	17.9	Yes
					SHV-12	31 (5.4)	3.9	5.4	7.5	No
	Imported	571	255	44.7	TEM-52	6 (1.1)	0.5	1.1	2.3	No
					Unknown	6 (1.1)	0.5	1.1	2.3	N/A
					CTX-M-2	5 (0.9)	0.4	0.9	1.9	No
					TEM-20	1 (0.2)	0.0	0.2	0.9	No
					Up-regulated AmpC	1 (0.2)	0.0	0.2	0.9	No
					SHV-2a	1 (0.2)	0.0	0.2	0.9	No
					Others	0 (0.0)	0.0	0.0	0.7	N/A

1 N/A is applied to ‘Unknown’ genotypes found in meat in DANMAP. It was not possible to determine which was the genotype detected.

2 N/A is applied to ‘Others’, since it may include genotypes that were present or not in human samples.

3 CMY-2 was detected in humans but its prevalence was not possible to calculate. However, it is known that few positive CMY-2 samples were detected. The total number of samples for each type of meat performed from 2009 to 2011 for DANMAP is represented by *n*, while s is the number of positive samples for each genotype in each type of meat. For a better understanding and comparison with the uncertainty analysis the genotype prevalence within each type of meat is shown.

The prevalence of CTX-M-1 was 0.2% in imported pork (23), but there was a high degree of uncertainty due to the low number of positive samples and total number of samples taken. The estimated upper limit of prevalence for this genotype was 3.8% in imported pork. CTX-M-14 was also estimated to be present in imported pork, with a prevalence below 1.3%. It should be noted that both CTX-M-1 and CTX-M-14 have been found in meat and in human cases in Denmark.

Imported broiler meat was the type of meat where the highest diversity of ESBL/AmpC genes was found in the DANMAP program. CMY-2 and CTX-M-1 had an upper limit of the estimated prevalence of 24.5 and 17.9%, respectively.

## Discussion

We evaluated the RC of various types of meat for the exposure of consumers to ESBL/AmpC-producing *E. coli* and their potential importance for human cases. We concluded that broiler meat was the type of meat that contributed the most to human exposure (83.8%), while CMY-2 was the genotype with the highest RC (58.3% of the total exposure through meat). However, this genotype has only been found on rare occasions in Danish human infections.

A definitive cause-effect association cannot be established through our approach. A genotype overlay is not proof that the sources were the actual route of exposure, so overlaps are indicative of a physical link between the source and the exposed individual that warrant further investigations. On the contrary, the absence of a genotype overlap, considering an appropriate sample scheme, provides evidence against a possible link. Therefore, inferences can be made with caution. With this in mind, the limited genotype overlap between the two reservoirs indicates that meat might play a minor role for human cases of ESBL/AmpC-producing *E. coli*.

### Data and methods

The meat consumption patterns were measured as the fresh and frozen meat available for consumption, not as the meat that was actually consumed. However, it was assumed that waste of meat occurred in the same proportion for all types of meat.

The bacterial capacity to survive in frozen and fresh meat might differ (27). However, data were not available to take the survival in different types of meat and storage types into account. Therefore, this might result in potential bias.

It was not possible to extend the study to other types of meat, such as turkey or horse meat, due to the lack of data on ESBL/AmpC-producing *E. coli* prevalences. Nonetheless, the presented exposure assessment covered the three types of meat that represent the vast majority of meat consumed in Denmark.

### The RC of meat to human exposure

Some changes were observed in the RC of each genotype from 2009 to 2011. Mainly minor shifts happened due to the sporadic detection of some ESBL/AmpC genes, and these were probably due to the limited sample sizes. The most evident change occurred with the increasing contribution of CMY-2 in Danish broiler meat from 4.7% in 2009 to 53.5% in 2011. This was due to the increasing prevalence of this genotype in Danish broiler meat, especially from 2010 to 2011 (23).

Considering that cephalosporins have not been used in poultry in Denmark for more than 10 years, it is possible that the origin of the problem is upstream in the production pyramid. This hypothesis has also arisen in Sweden (28, 29). Danish and Swedish broiler parents come from the same Swedish breeding stock, which in turn is supplied by a Scottish grand-parent breeding company, where cephalosporins have been used prophylactically until recently. In Denmark, an *all in/all out* policy and strict biosecurity measures are implemented. Nevertheless, transmission between flocks might be possible and should be dealt with: proper actions related to all logistics behind broiler feeding, technical support and broiler transportation to the slaughterhouses ((cages and trucks) which might help to limit the dissemination between flocks. Moreover, it should be investigated whether the animal feed might act as an important source of ESBL/AmpC-producing *E. coli*. However, the fact that CMY-2 and CTX-M-1 were also the genotypes detected in Swedish broiler meat (30) and both of them being the only genotypes persistently detected in Danish broiler meat from 2009 to 2011, supports the hypothesis of a common source upstream in the production pyramid. In contrast, imported broiler meat presented a substantial variety of genotypes (22–24), possibly due to the different importing countries and various causes of ESBL/AmpC emergence.

In general, the use of other antimicrobials in the broiler production may facilitate the spread and maintenance of ESBL/AmpC-producing *E. coli* through co-resistance patterns (29). Moreover, cross-contamination through the environment, humans and animals, as well as off-label use of cephalosporins are other ways of spreading resistance.

Denmark has one of the lowest antimicrobial uses in livestock of the EU and demonstrates a very prudent use of cephalosporins in animals (31). The discontinuation of the use of cephalosporins in 2010 in pigs is the most likely explanation for the reduction in the ESBL/AmpC-producing *E. coli* prevalence seen in pigs in 2011 (12). Other factors could also have contributed to this diminution, that is, the ‘Yellow card’ initiative, which took place almost at the same time and initially led to a 25% decrease in the use of antimicrobials in pigs (11, 32).

The homogeneity of the ESBL/AmpC-producing *E. coli* genotypes from imported beef is mostly likely a result of the low number of positive samples: one positive sample per year out of 298 samples analyzed. In Danish beef just two positive samples from different genotypes were found from 2009 to 2011 (data not published). The total number of Danish beef collected samples during the 3-year period was 398. Due to the low number of positive samples, beef is the type of meat in which the uncertainty about the true ESBL/AmpC genotype distribution is the highest.

### Genotype overlaps between meat and human infections

In humans, the so-called ‘pandemic’ CTX-M-15 genotype dominated in 2011 in Denmark, both in blood and urine ESBL-producing *E. coli* isolates, with 68.0 and 59.3%, respectively (23) (Fig. 3). CTX-M-1 was detected in 7.3% of urine and 8.0% of blood isolates (23). However, it should be noted that, particularly for human ESBL-producing *E. coli* septicemia, the number of cases that these results are based on is quite small (*n*=25). Consequently, it is possible that the true ESBL genotype distribution in Denmark is slightly different from what was found, and some genotypes may not have been detected.

ESBL/AmpC genes found in humans and meat were, as previously described, only congruent to a limited extent. Although some genotypes were not found in meat, they were found in live animals, indicating their possible presence in meat. That is the case of CTX-M-15, which was detected in very low prevalence in Danish pigs and Danish cattle. Its low prevalence probably explains why it was not detected in meat. This was one of the main motives that propelled us to perform an uncertainty analysis through the use of beta distributions. As expected, the mode prevalence was equal or very similar to the prevalence found in DANMAP and the uncertainty was higher with smaller sample sizes. The maximum expected prevalence for ESBL/AmpC genes not detected in meat, considering the DANMAP sampling within the 3-year period, was 0.7% for Danish pork, 0.9% for imported pork, 1.0% for Danish beef, 1.4% for imported beef, 0.9% for Danish broiler meat and 0.7% for imported broiler meat. Through our calculations, these are the upper 95% credibility limits for the expected prevalences for CTX-M-15 in each type of meat. Such low prevalence points against a relevant role of meat for the CTX-M-15-producing *E. coli* infections in humans. Nonetheless, more should be known about the duration of carriage, dominance in the human gut microflora and different reservoir adaptability and ESBL/AmpC-producing *E. coli*'s diverse ability to survive in the human gut environment.

CMY-2 was detected in human infections, but it was not possible to calculate its relative prevalence in urinary tract infections and septicemias due to lack of information. Nevertheless, the positive number of samples in humans was low (data not published). Considering the contribution of CMY-2-producing *E. coli* for human exposure through meat, its relative prevalence in human infections must be calculated in the upcoming years.

A quantitative exposure assessment to third-generation cephalosporin-resistant *E. coli* through the consumption of broiler meat performed in Belgium (33) also highlighted the lack of some important data to evaluate the impact of animal foodborne sources to human exposure.

### Control options

Our results suggest that meat does not have as high impact on human ESBL/AmpC-producing *E. coli* infections in Denmark as previously thought. This might be related to the actions taken in Denmark to limit the use of cephalosporins in livestock. Hence, this does not exclude the importance of animal production, and even meat, for ESBL/AmpC dissemination, so a precautionary approach should be taken.

In Denmark, there are limited additional options to control ESBL/AmpC occurrence through the reduction of the use of cephalosporins in livestock. However, other antimicrobials can co-select ESBL/AmpC-producing bacteria. Therefore, measures to promote the reduction of antimicrobial use in general, such as the ‘Yellow card’ scheme, may have some influence. Regarding the high ESBL/AmpC-producing *E. coli* prevalences in Danish broiler meat, measures have recently been taken to assure the non-use of cephalosporins in the top of the production pyramid (Jan Dahl, Danish Agriculture and Food Council, personal communication, 2013).

Moreover, actions taken to limit cross-contamination during slaughter should always be encouraged.

A judicious use of resources must be done. In that sense, the role of other sources should be assessed including the annual human consumption of cephalosporins. In 2009, the human consumption of cephalosporins in Denmark was 2,740 kg compared to 95 kg in pigs (22). This would allow us to point to the reservoir where most effort should be applied to control ESBL/AmpC-producing *E. coli*. Table 3 presents some general measures to control ESBL/AmpC-producing *E. coli* and ESBL/AmpC-producing bacteria in Denmark.

**Table 3:** Suggested measures to control ESBL/AmpC-producing bacteria in Denmark

Possible source	Measures
Human-to-human and nosocomial	Improve hospital hygiene
infections	Stricter guidelines, both for hospital and community use of cephalosporins
Animal-to-human transmission	Stricter guidelines for the use of cephalosporins in pets
Travelling	International measures to control ESBL/AmpC-producing bacteria
	Advice to travelers about hygiene measures to prevent becoming colonized with ESBL/AmpC-producing bacteria
Environment	Reduce the use of cephalosporins and antimicrobials in general; this should decrease ESBL/AmpC-producing bacteria dissemination
	Stricter evaluation of wastewater, including microbiological criteria and control of resistant bacteria
Foodborne	Avoid use of cephalosporins and reduce the use other antimicrobials to as little as possible but as much as necessary in livestock
	Production measures to reduce ESBL/AmpC-producing bacteria dissemination
	Improve slaughter hygiene
	Decontamination after slaughter

Meat might be the source for at least a small part of the human cases, but to obtain more precise conclusions, further studies are needed. ESBL/AmpC-producing bacteria is a complex subject and their emergence, ecology, and dynamics is nowadays poorly understood.

A wider study about the ESBL/AmpC genotypes in human cases in Denmark is recommended including an extensive characterization of human patients, that is, through a detailed and recorded patient history which may enable further investigations on the epidemiology of ESBL/AmpC-producing bacteria. Additionally, studying the risk factors for the colonization of healthy humans could be a relevant step for source attribution and important to prioritize control measures. However, new and more discriminatory molecular methods may be needed to distinguish different reservoirs and transmission routes.

The relevance of ESBL/AmpC-producing bacteria carriage for the occurrence of community acquired and nosocomial infections should also be assessed. This includes not only further investigations on the risk of transmission of ESBL/AmpC genetic material from bacteria in consumed food to commensal gut flora, but also an assessment of the relevance of ESBL/AmpC-producing bacteria carriage for the development of severe infections caused by the same bacteria.

## Conclusion

In this study, broiler meat was the type of meat with the highest RC for human exposure (83.8%), followed by pork (12.5%) and beef (3.7%).

We conclude that the genotype overlap between human and meat from poultry, pigs and cattle is low, suggesting that meat might not have such a relevant role for ESBL/AmpC-producing *E. coli* human infections in Denmark in 2009–2011, as previously thought. This is most likely related to the limited use of cephalosporins in Danish livestock.
